# Current Scientific Research Trends on Salivary Biomarkers: A Bibliometric Analysis

**DOI:** 10.3390/diagnostics12051171

**Published:** 2022-05-08

**Authors:** Beenish Fatima Alam, Talha Nayab, Saqib Ali, Rasha AlSheikh, Asim Mustafa Khan, Muntasar T. Al Hinai, Imran Farooq

**Affiliations:** 1 Department of Oral Biology, Bahria University Dental College, Bahria University of Health Sciences, Karachi 75070, Pakistan; nish_alam@yahoo.com; 2 Department of Dental Materials Science, Sindh Institute of Oral Health Sciences, Jinnah Sindh Medical University, Karachi 75070, Pakistan; talha.nayab@jsmu.edu.pk; 3 Department of Biomedical Dental Sciences, College of Dentistry, Imam Abdulrahman Bin Faisal University, Dammam 31441, Saudi Arabia; drsaqiibali@gmail.com (S.A.); amkhan@iau.edu.sa (A.M.K.); 4 Department of Restorative Dental Sciences, College of Dentistry, Imam Abdulrahman Bin Faisal University, Dammam 31441, Saudi Arabia; ralsheikh@iau.edu.sa; 5 Dental and Maxillofacial Surgery Department, Sultan Qaboos University Hospital, P.O. Box 35 Muscat, Oman; malhin7@gmail.com; 6 Faculty of Dentistry, University of Toronto, Toronto, ON M5G 1G6, Canada

**Keywords:** saliva, VOSviewer, scientiometrics, biomarkers

## Abstract

Salivary biomarkers are indicators of many biological and pathological conditions and provide further information regarding the early detection of diseases. This bibliometric analysis aims to identify and evaluate the scientific literature addressing salivary biomarkers from a dental perspective, to identify the most prolific organizations, authors, journals, countries, and keywords used within this research domain. An electronic search was performed using Elsevier’s Scopus database. From a total of 587 retrieved papers (published between 1997 and 2021), 399 were selected. For the data analysis and its visualization, the title of the articles, year of publication, countries, authors, journals, articles, and keywords were analyzed using Microsoft Excel and VOSviewer (a bibliometric software program). An increase in the number of publications was identified from 1997 to 2021. The United States (U.S.) published the most papers (84) and received the highest citations (3778), followed by India and Brazil. The *Journal of Periodontology* published the highest number of articles (39) that received the highest citations. The University of Kentucky from the U.S. published most of the papers related to salivary biomarkers that received the highest citations. Timo Sorsa published the most papers (14 papers), while Craig Miller was the highest cited author (754 citations). Concerning the highly cited papers, a paper by Micheal et al., published in 2010, received the highest citations (487 citations). “Saliva”, followed by “human”, were the most common keywords used by the authors in the papers related to salivary biomarkers. The findings of this analysis revealed an increase in salivary biomarker-related publications that positively influenced the number of citations each paper received. The U.S. produced the most publications that received the highest citations, and the University of Kentucky, U.S., was the most prominent. The articles were mostly published in the *Journal of Periodontology* and received the highest number of citations.

## 1. Introduction

Human saliva is a compound of various fluids and performs several functions, such as acting as a buffer to protect the teeth from acids, facilitating taste recognition, and containing various antimicrobial agents that modulate oral microbial flora [1]. Saliva entails huge diagnostic value, as it contains important inflammatory cytokines that are upregulated or downregulated in various diseases, and, thus, their levels can be checked to aid the early diagnosis of certain diseases [2]. However, it should be noted that salivary gland dysfunction can occur in certain diseases (such as diabetes mellitus), which could cause impairment of the saliva’s composition [3]. This may influence the presence or absence of inflammatory mediators related to other systemic conditions [3]. Therefore, although saliva could be used as a great diagnostic aid, the limitations associated with it should also be taken into account when making a clinical decision.

In terms of secretion, each salivary gland produces a secretion that differs in consistency, configuration, and components from the other salivary glands [4]. Major salivary glands are present on the posterior aspect of the oral cavity (parotid gland), the lower part of the mouth, in the middle of the cheek and jaw (submandibular gland), and are located inferolateral to the tongue (sublingual gland) [5]. Besides these, many minor secretory glands are present in the lip, cheek, tongue, and palate [6]. In addition, a small portion of fluid leaks from the area located between the gums and teeth, and it is composed of blood products that arise in the case of an injury to, or inflammation of, the mucosa. The amount of contribution from serum and saliva depends on the degree of mucosal or epithelial inflammation. Hence, “oral fluid” is a specific phrase used for all the fluid collected from the oral cavity, in which a substantial proportion is composed of saliva. Nevertheless, for clarity, this combination is identified as salivary biomarkers in this analysis [7].

The National Institutes of Health (NIH) stated that biomarkers could be accurately measured and act as indicators of normal biological development and pathological or pharmacological reactions to therapeutic involvement [8]. Biomarkers can provide specific information about the existing biological and functional state [9]. Biomarkers comprehensively detect therapeutical interventions, and specific information can be derived from their histological, molecular, radiographical, or physiological features [10]. These biomarkers can be present in diverse forms in the human body, including DNA, RNA, antibodies, lipids, proteins, and metabolites [11]. Variations in their function, concentration, and arrangement can be associated with the commencement, advancement, or even reversion of a specific disorder, or as a consequence of the body’s response to a disease [12]. Furthermore, a biomarker could be helpful in determining the incidence, site, and even probability of acquiring a disease [13]. Hence, biomarkers can serve as a valuable tool in the recognition, risk assessment, analysis, and prognosis of the disease [14].

Currently, salivary biomarkers are being used to diagnose various diseases. These biomarkers can be used to assess cortisol for Cushing’s disease and analyze stress-related conditions [15,16]. C-reactive protein (CRP), myoglobin, and creatinine kinase isoform can be used to assess heart diseases [17]; nucleic acids, pathogens, and antibodies for infections [18,19]; glycosylated hemoglobin (HbA1c) and α-2-macroglobulin for diabetes mellitus [20]; and interleukins (ILs) for the diagnosis of stomach diseases and muscle or joint disorders [21].

In the scientific literature, the number of citations can be used to assess the influence of research or a publication. The citation frequency is significant not only for the researchers and journals, but also for the institute where the research was conducted [22]. Currently, bibliometric analysis has turned into an accepted method to present the research patterns of the scientific literature [23]. It also provides evidence regarding the progress of a specific domain, accentuating the most relevant country, journals, authors, and institutes involved in the research area [24,25,26]. There is a deficit in the literature regarding the bibliometric analysis of salivary biomarkers. In addition, the main findings of this analysis could help researchers, academics, and students to characterize scientific results regarding salivary biomarkers, evaluate diagnostic strategies, and identify important topics and issues that will help design future research. For this reason, this analysis was carried out to identify and evaluate the scientific literature addressing salivary biomarkers from a dental perspective, to determine the most prolific organization(s), authors, journals, countries, and keywords used within this research domain.

## 2. Materials and Methods

### 2.1. Search Strategy

**Figure 1:**
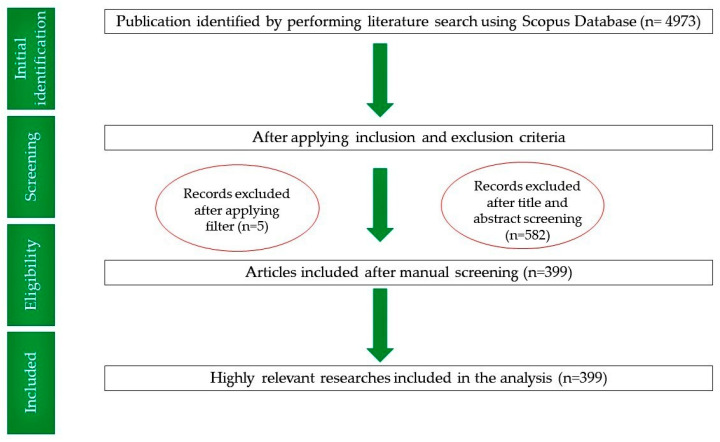
Four-phase flow diagram of data extraction and filtration process of publications related to salivary biomarkers.

For this analysis, an electronic search was performed in December 2021, using Elsevier’s Scopus database (https://www.scopus.com). The search subject was typed as “salivary biomarkers” and then added to the source title section. Authors carefully screened the titles and abstracts of prospective publications related to salivary biomarkers. From 582 retrieved papers, 399 were selected based on suitability (Figure 1). As this research did not involve any human or animal interaction, no ethical approval was obtained for this analysis.

### 2.2. Inclusion/Exclusion Criteria

The inclusion criteria for this bibliometric analysis included focusing on articles published in the English language only. Regarding the type of publication, “article” was selected, and “dentistry” was then chosen as the subject domain. The exclusion criteria comprised articles published in other languages and subject specialties other than dentistry. Throughout the initial screening process by the investigators, articles were further disqualified if the publications were not related to the domain of salivary biomarkers. As the research was conducted in December 2021, all the papers from 1997–2021 (the last 25 years) were counted, covering research related to salivary biomarkers.

### 2.3. Data Analysis

Following the selection of papers, data were exported from the Scopus database in the CSV format. Results from the search were then analyzed using VOSviewer. Tab-delimited files were further transferred to Microsoft Excel to formulate the tables. In the maps generated, the dimensions of the bubble showed the number of publications, while the distance between two bubbles exhibited the similarity between the two items. The color of each bubble had a distinct meaning in each visualization. Keywords with the highest number of occurrences were selected, and visualization maps were generated.

For data analysis and visualization, the title of articles, year of publication, countries, authors, journals, articles, and keywords were analyzed using Microsoft Excel and VOSviewer (v1.6.16; Centre for Science and Technology Studies, Leiden University, Leiden, The Netherlands), which is a bibliometric software program [27]. VOS viewer was used to generate a collaborative network for different variables and keywords.

## 3. Results

**Figure 2:**
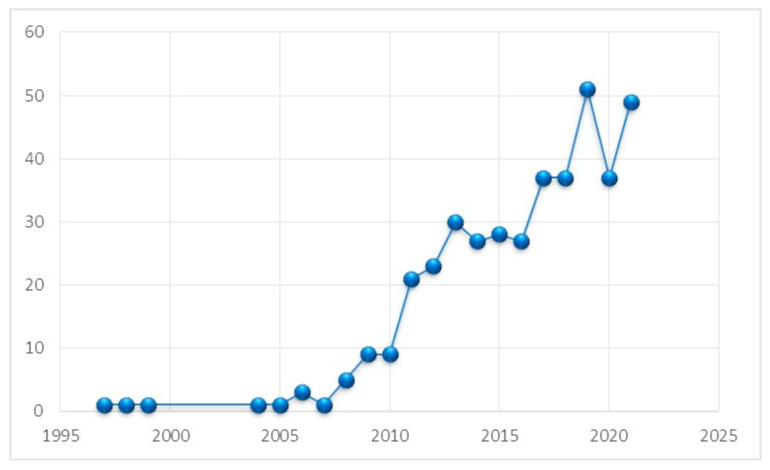
Total number of publications on salivary biomarkers from 1997 to 2021.

The results of our analysis revealed that from 1997 to 2010, the number of articles published on salivary biomarkers was relatively small (Figure 2). In 2011, 21 papers were published, while in 2012, 23 papers were published, showing that researchers developed a strong interest in this field. Similarly, in 2013, 30 papers were published, while in 2017 and 2018, 37 articles were published, respectively. The highest numbers of papers were published in 2019, with 51 publications, while in 2021 (up to December), 49 papers were published.

**Figure 3:**
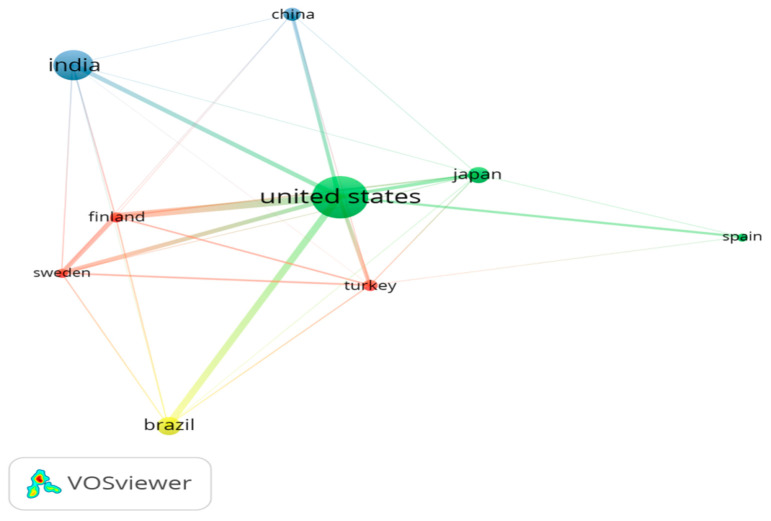
Showing the collaborative network of countries.

Table 1 identifies the leading countries that published the highest number of papers related to salivary biomarkers. The United States (U.S.) published the highest number of papers (84) and received the highest citations (3778), followed by India and Brazil, which published 60 and 36 articles, respectively. Concerning citations, India (1011) and Japan (627) received the highest citations after the U.S. (Table 1). In the current analysis, Figure 3 identifies a collaborative network among countries that collaborated for salivary biomarker-related research.

**Figure 4:**
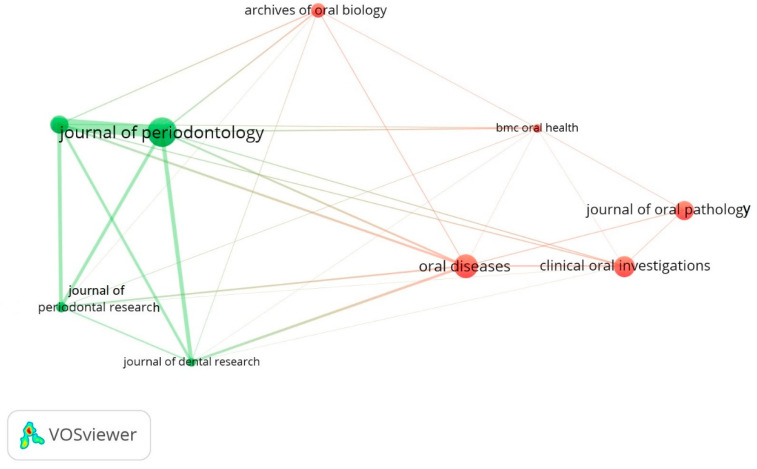
Showing leading journals that have published articles related to salivary biomarkers.

Table 2 exhibits the top journals that contributed to the domain of salivary biomarkers. The Journal of Periodontology published the highest number of articles (39) that received the highest citations (1247). The Journal of Oral Diseases and the Journal of Clinical Oral Investigations published 32 and 27 papers, while receiving 1083 (second highest) and 402 citations, respectively. Interestingly, the Journal of Clinical Periodontology only published 23 articles, but received 865 citations (third highest) (Table 2). In this analysis, Figure 4 illustrates the leading journals that have contributed to the domain of salivary biomarkers.

Table 3 displays findings of the leading institutes that published the most within the field of salivary biomarkers. The University of Kentucky from the U.S. published 9 papers, followed by the Ege University of Turkey and Universitas Indonesia, which published 6 papers each. The highest citations were received from the University of Kentucky (625), followed by the University of California and Ege University, receiving 301 and 162 citations, respectively (Table 3).

Table 4 identifies the leading authors who published papers related to salivary biomarkers. Timo Sorsa published 14 papers, followed by Craig Miller, who published 12 articles. At the same time, the authors Robert Jacobs and Tina Tervahartiala published 10 articles each. Craig Miller was the highest cited author (754), followed by Robert Jacob and William Giannobile, who received 745 and 567 citations, respectively (Table 4).

Table 5 displays the highly cited papers on salivary biomarkers. Among these, a paper by Micheal et al., published in 2010, received the highest citation (487) [28]. The subsequent most highly cited articles were by Sridharan et al., published in 2019, which received 266 citations, followed by Ramseier et al.’s paper, which was published in 2009 and received 240 citations [29,30] (Table 5).

In the present analysis, various keywords were used in the domain of salivary biomarkers. The top keywords found in this analysis, according to the level of occurrence, were saliva (318), human (301), female (205), biological marker (204), male (199), and biomarkers (147).

## 4. Discussion

To the best of the author’s knowledge, this is the first bibliometric analysis focusing on research based on salivary biomarkers published from 1997 to 2021 (the last 25 years). Several parameters, such as leading countries, organizations, journals, contributions of various authors, and keywords, have been analyzed with the help of bibliometric mapping. Bibliometric analysis helps to better visualize the organization and dynamics of scientific domains, to better understand a particular scientific field, and provide predictions regarding future trends [31].

The present analysis identified an increased number of publications from 1997 to 2021 (Figure 2), possibly as researchers identified the importance of salivary biomarkers as an essential indicator of the biological and pathological conditions that provide further information regarding the early detection of a disease [32]. The analysis results revealed that the U.S., India, and Brazil contributed the most to this field, with the U.S. publishing the highest number of papers and receiving the highest citations. A similar pattern of research from the U.S. has been observed in other scientific fields, for instance, dentistry, regenerative endodontology, endodontology, and implant dentistry [33,34,35]. Due to the enormous scientific community, in combination with the availability of funding opportunities by the National Institute of Dental and Craniofacial Research (NIDCR) in the U.S., the scientific community from this country performs cutting-edge research, resulting in highly impactful publications [36,37].

The University of Kentucky from the U.S. was the most prolific organization that published the highest number of papers, comparable to the previously conducted bibliometric analysis, where universities from the U.S. published the highest number of papers [38,39,40]. Interestingly, Craig S. Miller, followed by Robert Jacobs, both currently associated with the University of Kentucky in the U.S., were highly cited authors, identifying their role in producing highly impactful publications, making the University of Kentucky the leading organization in this domain. Nevertheless, Ege University from Turkey secured the second position, identifying an increase in publications from Turkey related to this domain. Similarly, Indonesian University published the third-highest number of papers, highlighting the rise in research within this field amongst the Southeast Asian countries, which agrees with the previously conducted bibliometric analysis [38].

An interesting finding of this bibliometric analysis was that the majority of the papers were published in the most influential and pertinent journals, having a high impact factor, reiterating the fact that papers published in these journals receive high citations [41,42]. In the current analysis, the *Journal of Periodontology* published the highest number of articles and received the highest citations. Additionally, publications produced due to international collaborations among the different countries produced research with higher impact and citation counts [43].

Regarding the most highly cited paper, it was published by Micheal et al. in 2010 [28]. In this article, the authors extracted exosomes from human saliva containing microRNAs, taken from the saliva of patients with Sjögren’s syndrome and controls. The microRNAs were further analyzed using real-time polymerase chain reaction (PCR) and microRNA microarrays, which proved valuable in identifying microRNAs [28]. The second most highly cited paper was by Sridharan et al., which discussed the identification of salivary metabolomics within patients with oral leukoplakia, squamous cell carcinoma, and controls, using mass spectrometry. This study proved useful in detecting tumor biomarkers that can be used for the prompt diagnosis and estimation of tumor progression [29]. The third highly cited article was by the author Ramseier et al., published in 2009. This study focused on identifying biomarkers using saliva collected from healthy and periodontitis patients, to identify the periodontal disease status from whole saliva and plaque biofilm. Quantitative PCR and immunoassays were used to measure the levels of multiple pro-inflammatory cytokines and bone resorptive/turnover markers associated with periodontal disease [30].

Various authors have assessed the role of salivary biomarkers with respect to periodontal disease. From the highly cited papers in our analysis, Miller et al. conducted a case–control study where IL-1β, MMP-8, and osteoprotegerin were identified as the biomarkers involved in periodontal disease [44]. Similarly, research by Kinney et al. [45] detected an association between periodontal pathogens and salivary biomarkers with periodontal disease. The authors identified that MMP-8, MMP-9, OPG, and IL-1β demonstrated a strong association with periodontal disease progression [45]. Another research paper by Sexton et al., published in 2011, also evaluated the role of various salivary biomarkers, namely, MMP-8, MIP-1α, OPG, and IL-1β, in periodontitis. The authors identified the positive role of salivary biomarkers in identifying the severity of diseases that could play a crucial role in identifying the periodontitis status [46]. Teles et al. aimed to identify the difference in the levels of 10 different cytokines present within healthy and periodontitis patients; however, no statistically significant association between the two groups was found [47]. Additionally, research by Rathnayake et al. also collected saliva samples for the detection of periodontitis using salivary biomarkers. The study findings revealed that participants with severe periodontitis had increased concentrations of IL-1β and MMP-8, while smokers also had slightly reduced concentrations of IL–8 and MMP-8 [48]. Lastly, Rai et al. compared the levels of MMP-2, MMP-9, and MMP-8 salivary biomarkers present in the GCF and saliva among patients with gingivitis and periodontitis, and healthy individuals. The study results identified a tremendous increase in the levels of MMP-8 and MMP-9, and a decrease in the level of MMP-2, in cases of periodontitis. Hence, the research identified the fact that MMP-8, MMP-9, and MMP-2 biomarkers can aid in the diagnosis of periodontitis [49].

Much scientific literature has been published to assess the role of salivary biomarkers in oral cancer and related conditions. One of the highly cited papers from our analysis (Li et al. [50]) identified 3000 various types of mRNA from the unstimulated and cell-free saliva, performed with the help of microarray technology, which can help to identify various oral and systemic diseases [50]. Similarly, Momen-Heravi et al. [51] also collected saliva to analyze salivary biomarkers in four different study groups, which included patients with oral squamous cell carcinoma (OSCC), OSCC in remission, oral lichen planus, and healthy individuals. The results identified an increased level of miRNA-27b in the OSCC, while miRNA-136 was under-expressed in other groups. Thirteen other miRNAs were also identified that were either deregulated or under-expressed [51]. A scientific paper by Segal and Wong discussed the beneficial role of salivary biomarkers in identifying oral cancer and other related conditions. Saliva can also be used for detecting other systemic conditions, such as diabetes, HIV, arthritis, and heart disease [52].

Keywords play an essential role in the discoverability of printed paper [53,54]. Generally, while performing a literature search, researchers tend to meticulously use the various search terms related to the specific domain [55]. In the current analysis, saliva, human, female, biological marker, male, and biomarkers were frequently used by the researchers as keywords in their publications.

Concerning the limitations of the current analysis, we only chose the Scopus database, and non-English articles were excluded. This can perhaps lead to selection biases. In addition, the method of data extraction was performed manually. Although the authors checked the database carefully, some mistakes might still exist, and readers should interpret the findings of this analysis cautiously.

## 5. Conclusions

It can be concluded, from this bibliometric analysis on salivary biomarkers, that an increase in the number of publications in recent years was noticed to positively influence the number of citations each paper received. Among the countries producing papers on salivary biomarkers, the U.S. published most of the articles that received the highest citations, and the University of Kentucky stood out amongst all other institutes. The articles were primarily published in the *Journal of Periodontology*, and the articles published in the same journal received the highest citations. Among the authors, Craig S. Miller received the highest citations, while Tim Sorsa published the highest number of papers. The most cited paper was credited to Michael et al., and it was published in *Oral Diseases*. The findings of our analysis could help researchers, academics, and students to characterize scientific results regarding salivary biomarkers, to evaluate diagnostic strategies and to identify important topics and issues that will help design future research.

## Figures and Tables

**Table 1:** Identifying the leading countries that published the highest number of papers related to salivary biomarkers.

Countries	Number of Articles	Citations	Total Link Strength
U.S.	84	3778	167
India	60	1011	37
Japan	31	627	39
Finland	20	522	83
Turkey	22	505	48
Brazil	36	382	48
Sweden	17	316	64
China PR	26	291	26
Spain	16	170	16

**Table 2:** Exhibiting the top journals that contributed to the domain of salivary biomarkers.

Journal	Number of Articles	Citations	Total Link Strength
Journal of Periodontology	39	1247	94
Oral Diseases	32	1083	42
Journal of Clinical Periodontology	23	865	89
Journal of Dental Research	11	704	43
Journal of Oral Pathology and Medicine	25	609	7
Clinical Oral Investigations	27	402	18
Archives of Oral Biology	18	363	17
Journal of Periodontal Research	13	343	41
BMC Oral Health	11	67	17

**Table 3:** Displaying leading institutes that published the most within the field of salivary biomarkers.

Institute	Country	Number of Papers	Citations	Total Link Strength
University of Kentucky, Lexington	U.S.	9	625	39
University of California, Los Angeles	U.S.	3	301	0
Ege University, Izmir	Turkey	6	162	1
University of Helsinki, Helsinki	Finland	4	157	10
University of Eastern Finland, Kuopio	Finland	3	100	8
Universitas Indonesia, Jakarta	Indonesia	6	9	0

**Table 4:** Identifying leading authors who published papers related to salivary biomarkers.

Author	Number of Articles	Citations	Total Link Strength
Craig S. Miller	12	754	155
Robert J. Jacob	10	745	144
William Giannobile	8	567	74
Jeffrey L. Ebersole	9	506	125
David T.W. Wong	8	438	13
Timo Sorsa	14	395	66
Taina Tervahartiala	10	202	39
Gülnur Emingil	7	164	20
Diana M. Isaza-Guzmán	7	128	15
Sergio I. Tobón-Arroyave	7	128	15
Nagihan Bostanci	7	108	14

**Table 5:** Displaying highly cited papers on salivary biomarkers.

Paper	Citations	Links
Michael, A.; Bajracharya, S.D.; Yuen, P.S.; Zhou, H.; Star, R.A.; Illei, G.G.; Alevizos, I. Exosomes from human saliva as a source of microRNA biomarkers. *Oral Dis.* **2010**, *16*, 34–38.	487	0
Sridharan, G.; Ramani, P.; Patankar, S.; Vijayaraghavan, R. Evaluation of salivary metabolomics in oral leukoplakia and oral squamous cell carcinoma. *J. Oral Pathol. Med.* **2019**, *48*, 299–306.	266	0
Ramseier, C.A.; Kinney, J.S.; Herr, A.E.; Braun, T.; Sugai, J.V.; Shelburne, C.A.; Rayburn, L.A.; Tran, H.M.; Singh, A.K.; Giannobile, W.V. Identification of pathogen and host-response markers correlated with periodontal disease. *J. Periodontol.* **2009**, *80*, 436–446.	240	3
Miller, C.S.; King, C.P., Jr., Langub, M.C.; Kryscio, R.J.; Thomas, M.V. Salivary biomarkers of existing periodontal disease: A cross-sectional study. *J. Am. Dent. Assoc.* **2006**, *137*, 322–329.	239	3
Li, Y.; Zhou, X.; St. John, M.A.; Wong DT. RNA profiling of cell-free saliva using microarray technology. *J. Dent. Res.* **2004**, *83*, 199–203.	182	2
Baliga, S.; Muglikar, S.; Kale, R. Salivary pH: A diagnostic biomarker. *J.* *Indian Soc. Periodontol.* **2013**, *17*, 461.	177	0
Kinney, J.S.; Morelli, T.; Braun, T.; Ramseier, C.A.; Herr, A.E.; Sugai, J.V.; Shelburne, C.E.; Rayburn, L.A.; Singh, A.K.; Giannobile, W.V. Saliva/pathogen biomarker signatures and periodontal disease progression. *J. Dent. Res.* **2011**, *90*, 752–758.	132	2
Sexton, W.M.; Lin, Y.; Kryscio, R.J.; Dawson, D.R., III, Ebersole, J.L.; Miller, C.S. Salivary biomarkers of periodontal disease in response to treatment. *J. Clin. Periodontol.* **2011**, *38*, 434–441.	129	3
Momen-Heravi, F.; Trachtenberg, A.J.; Kuo, W.P.; Cheng, Y.S. Genomewide study of salivary microRNAs for detection of oral cancer. *J. Dent. Res.* **2014**, *93*, 86S–93S.	117	0
Teles, R.P.; Likhari, V.; Socransky, S.S.; Haffajee, A.D. Salivary cytokine levels in subjects with chronic periodontitis and in periodontally healthy individuals: A cross-sectional study. *J. Periodontal Res.* **2009**, *44*, 411–417.	114	2
Rathnayake, N.; Åkerman, S.; Klinge, B.; Lundegren, N.; Jansson, H.; Tryselius, Y.; Sorsa, T.; Gustafsson, A. Salivary biomarkers of oral health–a cross-sectional study. *J. Clin. Periodontol.* **2013**, *40*, 140–147.	111	4
Rai, B.; Kharb, S.; Jain, R.; Anand, S.C. Biomarkers of periodontitis in oral fluids. *J. Oral Sci.* **2008**, *50*, 53–56.	106	1
Segal, A.; Wong, D.T. Salivary diagnostics: Enhancing disease detection and making medicine better. *Eur. J. Dent. Educ. Off. J. Assoc. Dent. Educ. Eur.* **2008**, *12*, 22.	102	1

## Data Availability

Not applicable.
